# Study protocol for identifying resting brain functional connectivity markers of response to continuous Theta Burst Stimulation and cathodal transcranial Direct Current Stimulation in patients with schizophrenia with persistent auditory hallucinations

**DOI:** 10.12688/wellcomeopenres.20956.2

**Published:** 2025-02-12

**Authors:** Vanteemar S. Sreeraj, Nishant Goyal, Sonia Shenoy, Srinivas Balachander, Shyam Sundar Arumugham, Rujuta Parlikar, Kiran Basawaraj Bagali, Uppinkudru Chithra, Preeti Sinha, Abhiram Narasimhan Purohith, Chandramouli Roy, Venkataram Shivakumar, Kaviraja Udupa, Kandavel Thennarasu, Umesh Shreekantiah, Samir K. Praharaj, Kesavan Muralidharan, Jagadisha Thirthalli, Urvakhsh Meherwan Mehta, Ganesan Venkatasubramanian

**Affiliations:** 1 Department of Psychiatry, National Institute of Mental Health and Neuro Sciences, Bengaluru, Karnataka, 560029, India; 2 Department of Psychiatry, Central Institute of Psychiatry, Ranchi, Jharkhand, 834006, India; 3 Department of Psychiatry, Kasturba Medical College, Manipal, Manipal Academy of Higher Education, Manipal, Karnataka, 576104, India, Manipal, Karnataka, 576104, India; 4 Department of Integrative Medicine, National Institute of Mental Health And Neuro Sciences, Bengaluru, Karnataka, 560029, India; 5 Department of Neurophysiology, National Institute of Mental Health And Neuro Sciences, Bengaluru, Karnataka, 560029, India; 6 Department of Biostatistics, National Institute of Mental Health And Neuro Sciences, Bengaluru, Karnataka, 560029, India

**Keywords:** Neuromodulation, non-invasive brain stimulation, head-on comparison, randomised controlled study

## Abstract

**Background:**

Schizophrenia is one of the most burdensome psychiatric disorders. Novel neuromodulatory techniques including cathodal transcranial direct current stimulation (c-tDCS) and continuous theta burst stimulation (cTBS) using repetitive transcranial magnetic stimulation (rTMS) are increasingly being used in alleviating the auditory verbal hallucinations that are persisting despite adequate antipsychotic treatment. Brain connectivity modulation by stimulating the left temporoparietal junction is thought to mediate these effects. The differential neurobiological mechanisms and efficacy of these two neuromodulation techniques are not known. We are unaware of any systematic studies that can guide clinicians in choosing between the two techniques. This protocol describes a parallel-arm, double-blind, randomized cross-over study to identify resting brain functional connectivity markers of response to cTBS and c-tDCS persistent auditory hallucinations to improve the efficacy of interventions.

**Methods:**

Two hundred and ten consenting patients with schizophrenia with persistent auditory hallucinations will be randomly assigned to receive 15 days (30 sessions) of c-tDCS+sham-TBS or sham-tDCS+cTBS over the left temporoparietal region at three study centres. After a gap of 2-4 weeks, non-responders to the intervention will cross-over to the other arm. Clinical assessments, neurocognitive assessments, and multimodal investigations (magnetic resonance imaging, electroencephalography, heart rate variability, investigative transcranial magnetic stimulation-transcranial direct current stimulation, gene polymorphisms) will be conducted at baseline and repeated after the end of both phases of the trial. A differential pre-treatment resting brain functional connectivity signal will identify responders to cTBS or c-tDCS.

**Registration:**

Clinical Trial Registry of India ( CTRI/2021/05/033783) registered on 25/05/2021.

## Plain Language Summary

This article describes the protocol of the study that aims to address the challenge of persistent auditory hallucinations in patients with schizophrenia. Specifically, in those where hallucination continues despite adequate antipsychotic treatment. The research focuses on two innovative neuromodulation techniques: cathodal transcranial direct current stimulation (c-tDCS) and continuous theta burst stimulation (cTBS) using repetitive transcranial magnetic stimulation (rTMS). Both methods target the left temporoparietal junction to alleviate auditory verbal hallucinations by modulating brain connectivity. However, the specific mechanisms and comparative superiority of these techniques is not known.

The study proposes a rigorous study design where 210 patients would receive both rTMS and tDCS, but with only one of them being true and the other sham. Participants will be randomly assigned to receive either c-tDCS with sham-TBS or sham-tDCS with cTBS for 15 days (30 sessions) at three study centres. Those who do not show improvement in hallucinations after 15 days of treatment will then cross over to the alternative intervention after a 2-4 week gap.

A detailed assessment of clinical outcome, neurocognitive tests, and various investigations, including magnetic resonance imaging, electroencephalography, heart rate variability, investigative transcranial magnetic stimulation-transcranial direct current stimulation, and gene polymorphism analysis, will be conducted at baseline and after both phases of the trial.

The key objective is to see if resting brain functional connectivity can predict a positive response to either cTBS or c-tDCS. Ultimately, the study aims to provide valuable insights to guide clinicians in selecting the most effective neuromodulation technique for alleviating persistent auditory hallucinations in patients with schizophrenia.

## Introduction

### Background rationale for the study

Schizophrenia is a complex neuropsychiatric disorder characterized by delusions, hallucinations, disorganized behaviour, and progressive cognitive deficits. This disorder is ranked among the top ten disabling medical disorders by the World Health Organization ( Collaborators, 2022). Despite the best available treatments, about 25 – 35% of patients with schizophrenia show partial or no clinical improvement ( Siskind *et al.*, 2017). Residual or persisting symptoms contribute to a critical component of the disability burden. In addition, most of the existing antipsychotic medications are associated with intolerable side effects as well.

Contextually, alternative paradigms involving neuromodulatory techniques are gaining increasing attention in the treatment of schizophrenia. Transcranial Direct Current Stimulation (tDCS) has been reported to help treat hallucinations resistant to antipsychotic treatment in patients with schizophrenia ( Bose *et al.*, 2018; Fregni *et al.*, 2021). tDCS is a non-invasive neuromodulatory technique that delivers low-intensity, direct current to cortical areas through the surface application of electrodes on the scalp, facilitating or inhibiting spontaneous neuronal activity ( Gowda *et al.*, 2019). Alternatively, several transcranial magnetic stimulation (TMS) paradigms have also been investigated for persistent auditory hallucinations. rTMS non-invasively administers repeated magnetic stimuli through the scalp to the underlying cortical regions to induce action potentials in these cortical neurons. Theta burst stimulation (TBS) is a well-tolerated resource-sparing protocol of TMS in which bursts of magnetic pulses are delivered in a short time. Uninterrupted bursts of magnetic pulses, as administered in Continuous TBS (cTBS), are known to induce long-term depression and reduce the excitability of the cortical neurons. cTBS is a safe and well-tolerated treatment option for auditory hallucinations when applied unilaterally ( Nieuwdorp *et al.*, 2015) as well as bilaterally ( Lefaucheur *et al.*, 2020; Tyagi *et al.*, 2022).

Auditory verbal hallucinations (AVH) are one of the most common symptoms in schizophrenia ( McCarthy-Jones *et al.*, 2017) and are specific target of interest with these neuromodulatory techniques. AVH are a major cause of distress and burden to patients with an increased risk of self-harm, suicide, violence or homicide ( Dugré & West, 2019; Kjelby *et al.*, 2015). A significant proportion of patients respond to antipsychotics but may persist in having distressing AVH even during the first episode of schizophrenia ( Sinkeviciute *et al.*, 2021). Even though the neuromodulatory effects of either TMS or tDCS have been investigated in many clinical conditions, there are only a few reported clinical trials in pain disorders in which these two interventions were compared head-to-head ( Andre-Obadia *et al.*, 2023; Bonifácio de Assis *et al.*, 2022; Forogh *et al.*, 2021). However, we are unaware of any published work that has compared cTBS and cathodal tDCS, both of which are known to have a modulatory effect on the temporoparietal junction (TPJ) in reducing hallucinations. The modulation of connectivity of brain networks is poised to be one of the major mechanisms mediating the alleviation of auditory hallucinations with both tDCS ( Mondino *et al.*, 2016; Paul *et al.*, 2022; Zhuo *et al.*, 2021) and cTBS. At present, there is no research study that informs whether a patient with schizophrenia may differentially respond to one of these two treatments (i.e. cTBS and cathodal tDCS); such information can be vital in expediting appropriate neuromodulation treatment choices. This research project addresses this compelling research question.

### Aims and hypothesis

***Primary***

To identify resting brain functional connectivity markers of response to continuous Theta Burst Stimulation (cTBS) and cathodal transcranial Direct Current Stimulation (c-tDCS) in patients with schizophrenia with persistent auditory hallucinations to improve the efficacy of interventions.
Hypothesis: A differential pre-treatment resting brain functional connectivity signal will identify responders to cTBS or c-tDCS

***Secondary***

- To compare the neurobiological profile of patients with schizophrenia with persistent auditory hallucinations with matched healthy controls using multi-modal data [brain imaging, Electroencephalography (EEG), TMS-tDCS perturbation study metrics, Heart Rate Variability (HRV), and neuroplastic gene polymorphisms].
- To examine the differential effect of cTBS versus c-tDCS on the neurobiological profile of patients with schizophrenia with persistent auditory hallucinations using multi-modal data (brain imaging, EEG, TMS-tDCS perturbation study metrics, HRV) and potential interactions with neuroplasticity gene polymorphisms.
- To evaluate the predictive utility of multi-modal data (brain imaging, EEG, TMS-tDCS perturbation study metrics, HRV, and neuroplastic gene polymorphisms) in identifying clinical response to cTBS or c-tDCS in patients with schizophrenia with persistent auditory hallucinations.

This study protocol adheres to the SPIRIT guidelines ( Sreeraj *et al.*, 2023) and the example consent form and study proforma can be found as *Extended data* ( Sreeraj *et al.*, 2023).

### Participants

***Trial design***

We employ a parallel-arm double-blinded randomized cross-over design with an allocation ratio of 1:1 to identify the differential pre-treatment resting-state functional connectivity marker of response to cTBS and cathodal tDCS in persistent auditory hallucinations in schizophrenia.

***Study setting***

This would be a hospital-based study. The study would be conducted on patients with persistent auditory hallucinations at three mental health care and research institutes in India- National Institute of Mental Health and Neurosciences (NIMHANS), Bengaluru; Central Institute of Psychiatry (CIP), Ranchi; and Kasturba Medical College (KMC), Manipal. The study would be centrally coordinated at NIMHANS.

### Selection of patients

Patients with schizophrenia (as per the selection criteria listed below) will be screened and recruited for this research study from the clinical services of the three participating institutes.

***Inclusion criteria***

1. Diagnosis of Schizophrenia spectrum and other psychotic disorders (DSM-5) ( APA, 2013);
2. Age 18–60 years;
3. Any sex;
4. Right-Handedness [Edinburgh Handedness Inventory] ( Oldfield, 1971);
5. Persistence of auditory hallucinations without remission despite treatment with at least one antipsychotic medication at an adequate dose for a minimum period of six weeks. Non-remission of auditory hallucinations is defined with a score of moderate or high (>2) on the Global Rating of Hallucinations in the scale for the assessment of positive symptoms ( Andreasen, 1984). Individuals who may have had received electroconvulsive therapy would also be included.
6. Capacity to consent for research studies as per the assessment using the University of California, San Diego Brief Assessment of Capacity to Consent (UBACC) ( Jeste *et al.*, 2007)
7. Written informed consent.

***Exclusion criteria***

1. Suicidal risk necessitating electroconvulsive therapy / any other psychiatric emergency
2. Score of > 6 on Calgary Depression Rating Scale Addington ( Addington *et al.*, 1994)
3. Pregnancy / Post-Partum state (within 6 months of childbirth) ( Chauhan & Tadi, 2022)
4. Substance use disorder in the last six months (except caffeine or nicotine)
5. Comorbid neurological/medical disease that can affect the brain structure/function
6. Any Contraindication for Magnetic Resonance Imaging (like metallic implants, claustrophobia)
7. Any Contraindication for Transcranial Magnetic Stimulation (assessed using TMS adult safety screen ( Keel *et al.*, 2001)
8. Any Contraindication for Transcranial Direct Current Stimulation

### Interventions

**Figure 1.**
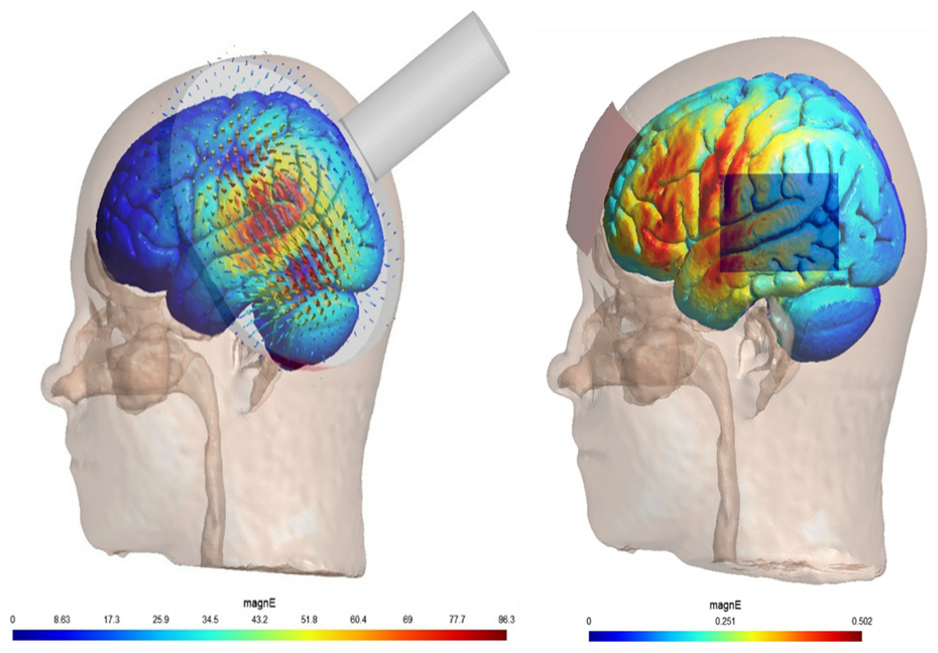
Demonstrates the simulated magnitude of the local electric field rTMS (using a standard Cool-B-70 coil for dI/dT of 66A/uSeconds) (on the left side) and conventional tDCS (for the parameters as described in the stimulation protocol above) (on the right side) for targeting persistent auditory hallucinations in patients with schizophrenia. The simulations were performed using SimNIBS ver 4.1 ( https://simnibs.github.io/) on the MNI-152 brain template. The colour gradient indicates the local magnitude of the electric field (magnE) in Volts/meter.

Participants would be randomised to receive either “cTBS with sham-tDCS” or “active tDCS with sham TBS.” The stimulation will be administered after localising the left TPJ based on the anatomical localisation method using T1-weighted MRI, wherever available. The MR image in the Anterior Commissure-Posterior commissure (AC-PC) plane would be 3D reconstructed. The junction where the left Sylvian fissure takes an upward and/or backward turn would be identified. An imaginary plane would be drawn in the coronal section cutting across this junction. The deepest point on the superior temporal sulcus in this imaginary coronal plane would be the target point for stimulation ( Dollfus *et al.*, 2018). When the T1-Weighted MR image is unavailable, the junction between anterior 1/4th and posterior 3/4th in an imaginary line drawn joining T3 and P3 located using 10–20 EEG system will be considered left TPJ for targeting the stimulations. The electric field modelling of the TMS and tDCS is provided in Figure 1.

***Continuous Theta Burst Stimulation (cTBS) with sham tDCS***

cTBS will be delivered to the left temporoparietal junction (TPJ) as triplet 50 Hz bursts, repeated at 5-Hz (given every 200 ms); in a continuous train of 40 s, repeated thrice at intervals of 5 min for a total of 1800 pulses per session; at 120%RMT. Neuronavigated-TMS would be administered for cTBS wherever available.

Sham tDCS would be administered using a cathode placed over the left TPJ and an anode placed over the left dorsolateral prefrontal cortex (DLPFC). The left DLPFC would be identified as the scalp location midway between F3 (determined using Beam F3 software) and Fp1 of 10–20 EEG system. Conductible rubber electrodes would be placed inside 0.9 mmol saline wetted 5×7cm pouches ( https://soterixmedical.com/) for delivering tDCS. In sham stimulation, a direct current would be administered with 20 seconds of fade-in incrementing to 2mA intensity, followed by 20 seconds of fade-out phase. There will be no current delivery during the rest of the 20-minute duration.

***c-tDCS + Sham-TBS***

In active tDCS, the electrode placement and stimulation parameters for sham-tDCS would be maintained as mentioned above. A 2mA current will be delivered for 20 minutes with 20 seconds each of fade-in and fade-out periods ( Bose *et al.*, 2018).

For sham TBS - intermediate theta-burst stimulation paradigm (im-TBS) would be administered using the targeting protocol as mentioned for cTBS. The im-TBS bursts would consist of 5-s theta burst trains repeated every 15 s (ie.,10 seconds inter-train interval) ( Bagali *et al.*, 2023). A train of 225 pulses (35 secs) will be repeated thrice every 5 mins. Two such sessions will be delivered each day, spaced across at least 3 hours. The im-TBS protocol has little effect on the underlying cortical excitability ( Bagali *et al.*, 2023) and has been used as a sham protocol in previous TBS clinical trials ( Kohutova *et al.*, 2017). Since true TMS pulses will be delivered, the risk of participant bias will be minimal.

Two such sessions will be delivered each day, spaced across at least 3 hours for 15 days for a total of 30 sessions. The TBS and tDCS (active or sham) will be delivered one after the other in the same order for a given subject throughout the course of the intervention. The order of TBS and tDCS will be altered in consecutive subjects at each site.

Adherence to intervention

All sessions would be provided at the hospital by a team of researchers. The financial burden on the participants would be mitigated by covering the incidental expenses and reasonable compensation for their time and other costs. Adverse effects would be diligently monitored, and any discomfort to participants would be addressed. In case of intolerance to TBS, the highest tolerable dose below 120%RMT will be administered. The research subject will have easy access to the team of investigators to clarify any study-related queries. These measures would enhance intervention adherence and study retention.

Concomitant care

In case of any deviation from the study guidelines due to unforeseen situations, the same would be documented and reported. Participants with a change in antipsychotic dose of more than 25% from the dose at the initiation of the intervention or a change in the antipsychotic medication or other neuromodulatory interventions would be considered drop-outs. All ongoing psychotropic medications and psychosocial interventions would be allowed to be concomitant with the study intervention. No new interventions would be initiated during the study.

Outcome

The primary outcome measure would be a differential change in the resting-state functional brain connectivity after “cTBS+sham tDCS” vs “active tDCS+ im-TBS”. Functional MRI will be acquired at baseline and after the end of each step of the intervention. Identifying the resting state markers would partake seed-based connectivity approach around the stimulation-targeted as well as other hallucination related brain region including fronto-temporo-parietal opercular regions, insula, angular gyrus, hippocampus and parahippocampal gyri. ( Jardri *et al.*, 2011b; Kühn & Gallinat, 2012; Rollins *et al.*, 2019; Thomas *et al.*, 2016; van Lutterveld *et al.*, 2013; Zmigrod *et al.*, 2016)

Secondary outcome measures include broader clinical symptoms, adverse effects, and neurobiological parameters (The detailed protocol of neurobiological parameters will be described in a separate paper). Clinical symptoms would be measured by the auditory hallucinations rating scale (AHRS) ( Haddock *et al.*, 1999), Scale for assessment of positive symptoms (SAPS) and negative symptoms (SANS) ( Andreasen, 1983; Andreasen, 1984), Calgary Depression Scale for Schizophrenia (CDSS) ( Addington *et al.*, 1994), Clinical Global Impressions (CGI) and Montreal Cognitive Assessment (MoCA) ( Nasreddine *et al.*, 2005) will be applied at baseline and every week till the end of the intervention. Cognitive and functional abilities will be assessed using the Brief Assessment of Cognition in Schizophrenia (BACS) ( Keefe *et al.*, 2004), Groningen Social Disability Schedule (GSDS) ( Wiersma *et al.*, 1988) and social and occupational functioning scale (SOFAS) ( Morosini *et al.*, 2000) at baseline and after each step of intervention.

Safety of the intervention sessions will be another secondary outcome measure: the tDCS-related adverse effect questionnaire Field ( Brunoni *et al.*, 2011; Chhabra *et al.*, 2020) and the TMS-related adverse effects questionnaire Field ( Giustiniani *et al.*, 2022) will be used to monitor adverse effects after each intervention session.

### Participant timeline

Step1: Patients meeting the selection criteria would be approached to participate in the study. A written informed consent would be obtained as approved by the ethics committees, after describing the study procedures with video aids. All treatments would be added to ongoing pharmacotherapy. In step-1, Participants would be randomised to either of the groups to receive “cTBS with sham-tDCS” or “active tDCS with sham TBS” twice a day for 15 days (over 3 weeks).

Step-2:

Upon non-response to step-1, participants would cross over to the other set of interventions in step-2 after a wash-out period of 2–4 weeks. Participants with <30% improvement in AHRS from baseline at the end of 2–4 weeks of the trial would be considered non-responders for inclusion to Step-2. Written informed consent would again be obtained before the initiation of step 2.

**Figure 2.**
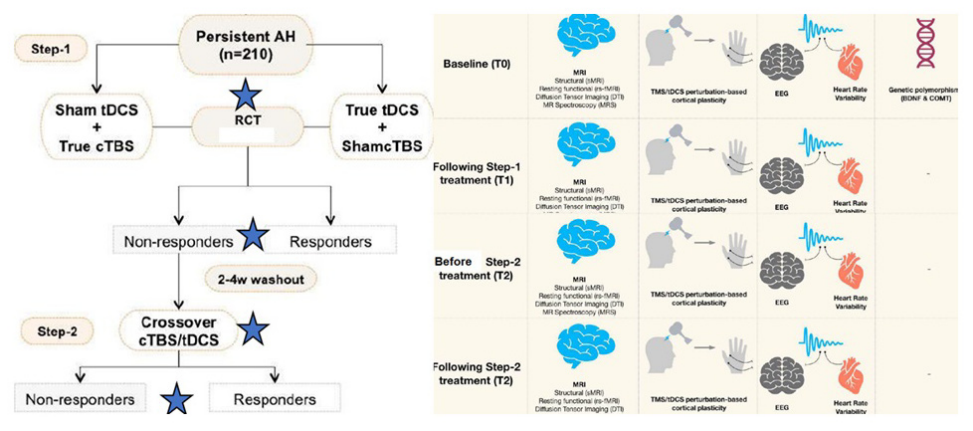
Depiction of the study procedure. In the first step of the study, the patients with persistent auditory verbal hallucinations would be randomized to receive 15 days of cTBS+sham-tDCS or sham TBS+active tDCS. Patients with clinical non-response even after 2–4 weeks of the end of the trial will be cross-covered to receive 15 days of the other set of interventions. Neuroimaging, and neurophysiological tests would be done before initiating and after the end of intervention with each step. Blood samples would be collected for genetic polymorphism assays at the baseline. Blue stars depict the time points of symptom rating and multimodal data acquisition. SZ: Schizophrenia, tDCS: transcranial direct current stimulation, cTBS: continuous theta burst stimulation, TMS: Transcranial Magnetic Stimulation, MRI: Magnetic resonance imaging, EEG: Electroencephalogram, TMS/tDCS: Transcranial magnetic stimulation/transcranial direct current stimulation, BDNF: Brain-derived neurotrophic factor, COMT: Catechol-O-methyltransferase.

The order of TBS and tDCS would be maintained as was delivered in step-1 for the given participant (See Figure 2).

***Sample size***

In order to identify a clinically relevant and appropriately corrected statistically significant signal from resting-state brain connectivity data with sufficient (~80%) power to differentiate (P<0.05) groups of responders and non-responders, a sample size ranging from 27 to 32 has been recommended for each group ( Pajula & Tohka, 2016; Thirion *et al.*, 2007; Zandbelt *et al.*, 2008) and similar sample size has shown utility in studies attempting to predict response ( Khalili-Mahani *et al.*, 2017; Martens *et al.*, 2022; Parlikar *et al.*, 2023; Zhang *et al.*, 2022). Keeping with this, we will recruit 210 participants who will be randomized in part-1 of the treatment. Anticipating ~15% dropout, we will have ~180 participants who will complete the part-1 treatment. Assuming a third of the participants respond to either treatment arm, a sample of 60 and 120 will be categorised as responders and non-responders, respectively. We expect at least three-quarters of the non-responders to consent to the crossover treatment. Given our experience in a previous trial ( Bose *et al.*, 2018), this will result in about 90 participants receiving the crossover treatment in part-2. A sample of 210 recruited at baseline will provide us with a minimal sample of ~30 participants in each group to permit sufficiently powered statistical inference about resting-state brain connectivity determinants of treatment response. Appropriate statistical modelling will be performed to delineate resting brain connectivity networks to identify general and specific response signals to cTBS and c-tDCS. For comparison studies between patients and healthy controls, a sample size of at least 210 healthy subjects will offer optimal power as per the sample size of previous neurobiological studies in this area.

### Allocation

***Sequence generation***

Consenting patients would be randomly assigned using computer-generated codes to true/sham interventions. Random allocation of 2 treatments (iTBS+sham-tDCS and active tDCS+sham-TBS) for 3 study centres with unequal strata sizes of 126, 42 and 42 would be carried out in Stata Statistical Software release.15.1 (College Station, TX: StataCorp LLC). This procedure would provide a sequence list, which would be randomly permuted in blocks of varying sizes and order. An independent statistician would manage the randomisation and allocation concealment. The allocation sequence generation is centrally coordinated at NIMHANS.

***Concealment mechanism***

Allocation concealment would be ensured through sequentially numbered sealed opaque envelopes.

***Blinding (masking)***

The participants, their caregivers, investigators, safety assessors, outcome assessors, treating psychiatrists, data managers, and data analysts would be blinded to the allocated study arm.

***Emergency unblinding***

Serious adverse events (SAE) would be reported to the trial management group (TMG) by the site investigator, and the principal investigator would thereafter report it to the data and safety monitoring board (DSMB), which would assess for suspected unexpected serious adverse reaction (SUSAR); the patient, caregiver, clinical team, and the investigators may be unblinded at their discretion. Assessment of blinding efficacy would be done on patients and outcome assessors after the last (scheduled/terminated) day of intervention with each step using a 5-point Likert scale.

***Trial procedures and evaluations***

In addition to the above-discussed screening and outcome tools, all participants would be assessed using a comprehensive semi-structured proforma to collect the socio-demographic and clinical details. Mental health professionals would perform clinical ratings. They would be trained in administering the clinical rating scales, and inter-rater reliability assessments would be performed for outcome measures. Comprehensive data collection forms would be available with the investigators and would be made available on reasonable request.

Multi-modal neurophysiological data (magnetic resonance imaging, EEG, cortical perturbation using transcranial magnetic stimulation- transcranial direct current stimulation, heart rate variability) would be acquired before and after interventions to identify predictive factors and understand the mechanistic basis of the treatment outcomes.

MRI data will be acquired in a 3T scanner (Ingenia CX, Philips). Structural MRI: T1-weighted single-shot 3D turbo field echo image.TR=2500-ms; TE=2.9-msec; FOV=256-mm; slice-thickness=1-mm; Matrix=256*256; Voxel size= 1*1*1-mm. Resting-state fMRI (RS-fMRI): Participants will be instructed to relax, not think anything actively, and lay still with their eyes open. The scan will be acquired with the following parameters: TR=2200 ms; TE=28-ms; slice-thickness=3mm; slice-number=44; Gap=0.3mm; Matrix=64*64*64; voxel=3.0-mm isotropic with 275-dynamic scans that lasts for 10 minutes and 20 seconds.

**Figure 3.**
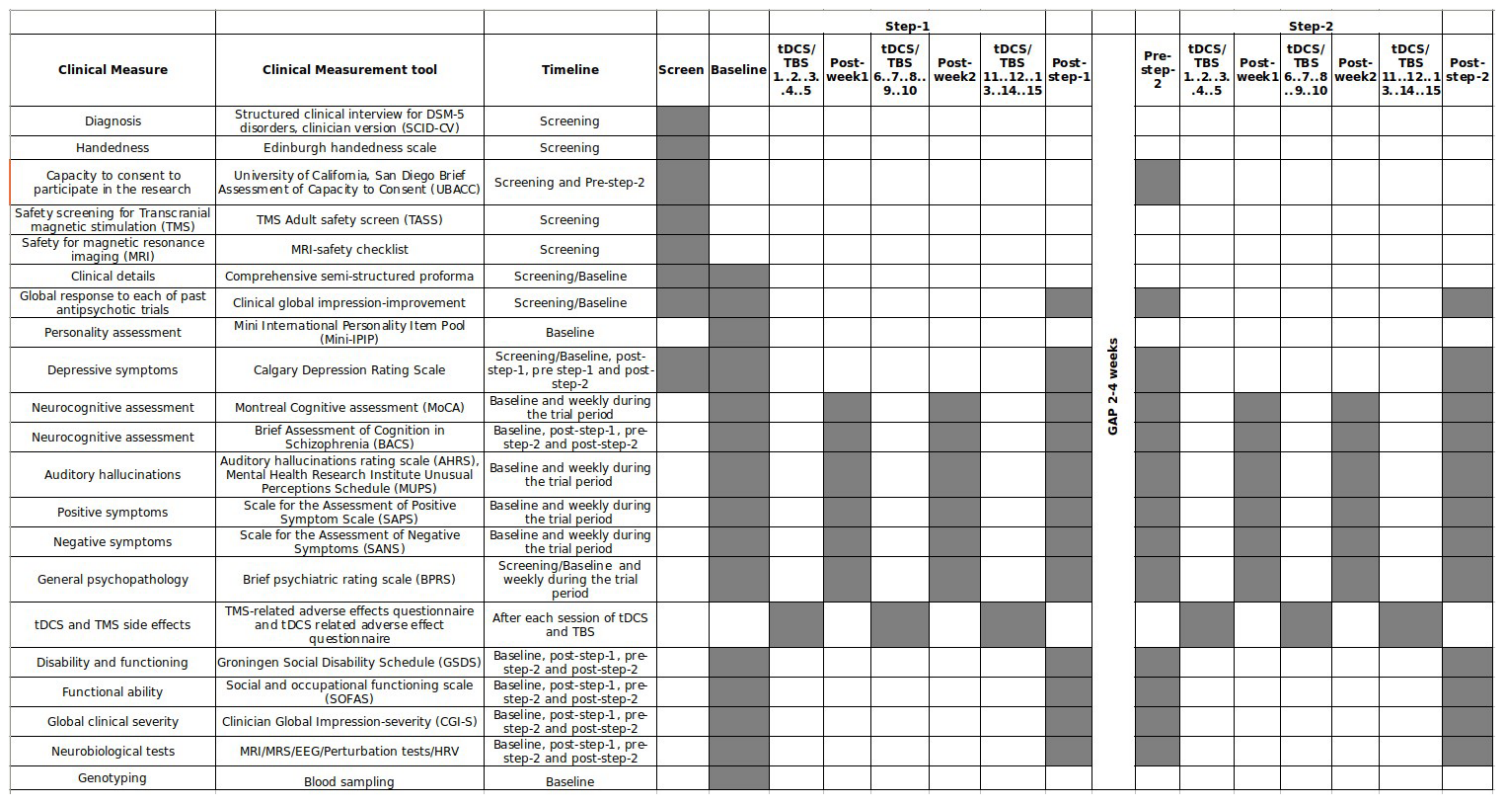
Gantt chart of the assessment timeline in patients. Grey boxes are scheduled timepoints of respective assessments.

Clinical and neurobiological evaluations would be repeated before and after each intervention step (Detailed in Table 1 and Figure 3). Also, neuroplasticity gene polymorphisms (Brain-derived neurotrophic factor (BDNF) and catechol-O-methyltransferase (COMT) genes) will be assessed alongside clinical and neurophysiological data to identify the predictors of clinical response. At least 300 subjects matched (as a group) for age, sex, education, handedness, and socio-economic status would be chosen for the comparative analyses of neurobiological studies. They would undergo a one-time baseline assessment of clinical and neurobiological parameters. The details of the neurobiological data acquisition protocol will be described in a separate paper.

**Table 1.** Scheduled Clinical assessments in the study.

Clinical Measure	Clinical Measurement tool	Timeline
Diagnosis	Structured clinical interview for DSM-5 disorders, clinician version (SCID-CV)	Screening
Handedness	Edinburgh handedness scale	Screening
Capacity to consent to participate in the research	University of California, San Diego Brief Assessment of Capacity to Consent (UBACC)	Screening and Pre-step-2
Safety screening for Transcranial magnetic stimulation (TMS)	TMS Adult safety screen (TASS)	Screening
Safety for magnetic resonance imaging (MRI)	MRI-safety checklist	Screening
Clinical details	Comprehensive semi-structured proforma	Screening/Baseline
Global response to each of past antipsychotic trials	Clinical global impression-improvement	Screening/Baseline
Personality assessment	Mini International Personality Item Pool (Mini- IPIP)	Baseline
Depressive symptoms	Calgary Depression Rating Scale	Screening/Baseline, post-step-1, pre step-1 and post-step-2
Neurocognitive assessment	Montreal Cognitive assessment (MoCA)	Baseline and weekly during the trial period
Neurocognitive assessment	Brief Assessment of Cognition in Schizophrenia (BACS)	Baseline, post-step-1, pre-step-2 and post-step-2
Auditory hallucinations	Auditory hallucinations rating scale (AHRS), Mental Health Research Institute Unusual Perceptions Schedule (MUPS)	Baseline and weekly during the trial period
Positive symptoms	Scale for the Assessment of Positive Symptom Scale (SAPS)	Baseline and weekly during the trial period
Negative symptoms	Scale for the Assessment of Negative Symptoms (SANS)	Baseline and weekly during the trial period
General psychopathology	Brief psychiatric rating scale (BPRS)	Screening/Baseline and weekly during the trial period
tDCS and TMS side effects	TMS-related adverse effects questionnaire and tDCS related adverse effect questionnaire	After each session of tDCS and iTBS
Disability and functioning	Groningen Social Disability Schedule (GSDS)	Baseline, post-step-1, pre-step-2 and post-step-2
Functional ability	Social and occupational functioning scale (SOFAS)	Baseline, post-step-1, pre-step-2 and post-step-2
Global clinical severity	Clinician Global Impression-severity (CGI-S)	Baseline, post-step-1, pre-step-2 and post-step-2
Neurobiological tests	MRI/MRS/EEG/Perturbation tests/HRV	Baseline, post-step-1, pre-step-2 and post-step-2
Genotyping	Blood sampling	Baseline

***Data management***

The multi-level data acquired from the enrolled participants would be quality-checked and de-identified to remove subject identifiers. These data would be stored with a coded identification. Different streams of data (ex. Clinical, MRI, EEG etc.,) would be linked using codes to facilitate seamless retrieval of different data types.

### Data analysis plan

Anonymized, coded data will be used for analysis. Pre-processing (where applicable – MRI/EEG data) will be done as per internationally accepted processing pipelines. Data analysis will be carried out in two phases – a) association and b) prediction. The first phase will involve exploratory analyses with biographic (age, sex, etc.) and disorder-specific (age-at-onset, medication, the severity of symptom, etc.) features that influence TMS-derived neuroplasticity metrics, MRI, EEG, HRV, and gene polymorphism measures. These include traditional bio-statistical procedures such as descriptive statistics and hypothesis testing for associations in order to identify any potential biomarkers of clinical response. Evaluation of clinical outcomes will be based on Last-observation carried forward analysis with secondary intent-to-treat analysis. The second phase of analysis will involve building predictive models that will be validated with an independent dataset. Here, instead of examining which individual measurements correlate with outcome measures, we seek to identify combination features (from resting-state fMRI as the primary objective; combining across data modalities will be the secondary objective) that can give the best prediction for the clinical outcome of proposed brain stimulation methods.

State-of-the-art feature engineering/machine learning methods such as gradient boosting/deep neural nets will be used to train the model on a part of the acquired dataset. Lastly, causal models using Bayesian networks or mediational analyses will be explored to uncover potential causal relationships between study variables.

***Data monitoring***

Formal committees

The data safety monitoring board, an independent advisory body, assesses data during the course of a study contributing to the scientific and ethical integrity of the study. It would periodically review and evaluate clinical efficacy and safety data collected during the study and assess reports on cumulated SAE. It consists of a biostatistician, psychiatrist, and ethicist specializing in psychiatry. Based on the DSMB charter, the board would provide a recommendation report on the continuation, modification, suspension, or termination of the trial to the study sponsor. The sponsor would submit the report to the ethics committees and trial management group (TMG) to decide on a further course of action.

TMG would comprise a group of investigators from the three collaborating institutes. It would monitor the day-to-day execution of the trial. It would take cognizance of trial participant drop-out in case of withdrawal of consent, non-compliance with study guidelines, and worsening of symptoms. It would interact with the DSMB and IEC. It would review the reports and recommendations of these oversight/monitoring committees and plan appropriate actions. It would decide on trial terminations and any trial modifications of the study procedures. It would also oversee the submission of modification, premature termination (if any), and final (completed) trial summary report to the IEC, trial registries, and funding agency.

The data management committee (DMC) comprises the principal investigators of the three institutes. It would be dedicated to reviewing the data archival, management, and sharing. It would execute the periodic data auditing and audit of the research practices related to the studies. It would ensure anonymization and de-identification of data. It would monitor the storage of data in the encrypted data storage servers. It would oversee the sharing of de-identified data upon reasonable requests from collaborators or the research community. Data sharing will be implemented as per the policy of DBT- Wellcome Trust India Alliance and International Good Research Practices in strict compliance with the ethics guidelines for biomedical research in India.

### Safety and harm

At no point in the study participants would be exposed to placebo treatment. That is to say; both groups would receive an active treatment during step-1 and step-2. Safety considerations have been thoroughly evaluated, and scientific rationale for the stimulation parameters within safety norms has been proposed. Also, during any of these interventions (tDCS and cTBS), both the subject and the doctor can talk to each other. Very rarely, some people may feel uncomfortable. If the subject develops significant discomfort during any assessment/intervention, the procedure will be immediately aborted.

### Safety of the procedures

***tDCS***

Over the past years, as a part of earlier/ongoing research studies that the NIMHANS ethics committee has approved, tDCS has been administered to patients with schizophrenia as well as patients with several other psychiatric diagnosis (for example, OCD) (about 150+ patients have received tDCS over the past years with a total number of patient-sessions more than 2000) using standard equipment as per established guidelines with stringent safety measures with evaluation using a comprehensive checklist after each session. These sessions were well tolerated, and there were no significant adverse events ( Chhabra *et al.*, 2020). This is in tune with the systematic review that examined data from about 209 studies and concluded that tDCS is a safe technique ( Brunoni *et al.*, 2011).

***TMS – cTBS***

Since administering low dose theta-burst stimulation was not found beneficial in treating persistent auditory hallucinations, we propose to increase the dose by (a) increasing the strength of stimulation to 120% of the resting motor threshold as used in a previous ( Loheswaran *et al.*, 2017) study and (b) deliver 1800 pulses as used in the ( Li *et al.*, 2014) study, but spaced out every 5 minutes. This protocol has been used at our centre in about ten patients with persistent auditory hallucinations without any significant adverse event, including seizures. Increasing the stimulation strength (110% of resting motor threshold) of theta-burst stimulation in combination with increasing the number of pulses administered per session (1620 pulses per session repeated five times a day in an accelerated fashion) has also been in practice in accelerated theta-burst protocols in depression, which have been found to be safe and well-tolerated ( Desmyter *et al.*, 2016). Similarly, continuous theta-burst has also been delivered at 100% of the resting motor threshold for 800-pulse trains and repeated four times a day ( Nyffeler *et al.*, 2019). Given the resistant nature of symptoms and favourable tolerability of 120% resting motor threshold and 1800 pulses stimulation (spaced out), we believe that modifying the stimulation parameters as done here is necessary to drive therapeutic benefits without compromising safety and tolerability.

This cTBS protocol is within the existing safety guidelines ( Loheswaran *et al.*, 2017; Oberman *et al.*, 2011; Rossi *et al.*, 2009; Rossi *et al.*, 2021) and is considered to be of more than minimal risk, with very few minor (scalp discomfort) or major (seizures) risks. Patients would be screened for TMS adult safety Screen (TASS) before recruitment, and TMS-related adverse effects would be monitored after each session using a questionnaire ( Giustiniani *et al.*, 2022).

 SAE, if any, would be reported to the trial management group (TMG) and data and safety monitoring board (DSMB), which would assess for suspected unexpected serious adverse reaction (SUSAR). In the unlikely event of harm attributable to participation in research procedures, financial compensation would be provided as per the National Ethical Guidelines for Biomedical and Health Research involving Human Participants by the Indian Council of Medical Research (ICMR).

Ancillary care and post-trial care

In the event of withdrawal of consent, non-compliance with study guidelines, and worsening of symptoms, the concerned participant would be dropped from the trial. The treating teams of the patients would provide the best-practiced standard care at the respective institutes even after the trial.

Auditing

The trial management group and data management committee would periodically audit data and the research practices. They would ensure the quality of research and patient advocacy.

## Ethics and dissemination

The research protocol would be implemented in strict adherence to the National Ethical Guidelines for Biomedical and Health Research involving human participants ( Mathur & Swaminathan, 2018) by the Indian Council of Medical Research and Declaration of Helsinki. The team of investigators has rich experience in all the research procedures. We would implement all the research procedures taking utmost care to avoid any injury. In the unlikely event that the research subject suffers any injury attributable to participation in the research procedures of this project, the subject would be compensated financially as per the National Ethical Guidelines for Biomedical and Health Research involving Human Participants by the Indian Council of Medical Research. Required insurance coverage would be obtained using the research funds from the funding agency - DBT Wellcome Trust India Alliance. The funding agency is in alignment with this policy of insurance coverage. The subject is free to withdraw the consent at any time during the study without giving a reason. If the subject is a patient, a decision to withdraw or not to take part would not affect routine medical care provided at NIMHANS.

All the tests and procedures carried out for the research purpose would be free of charge. The confidentiality of research data is ensured. In the case of data sharing, only de-identified data would be used as per the regulatory guidelines & international standards of practice. None of the researchers/investigators affiliated with this project or clinical research centre have any financial or non-financial (personal, academic, or political) conflict of interest to declare.

Ethics approval: Requisite clearances have been obtained from the ethics committees of all three institutes (i.e., NIMHANS, CIP, KMC) and from an independent ethics committee not affiliated with any of the three institutes (MS Ramaiah Medical College Ethics committee). (IEC approval numbers: Institute ethics committee of Central Institute of Psychiatry (CIP) approval: No.IEC/CIP/2020-21/337 dated 22/05/2021; Behavioural sciences division of National Institute of Mental Health and Neurosciences (NIMHANS) Institute Ethics committee approval: NIMHANS/EC (BEH.SC.DIV.) Institute ethics committee of Kasturba Medical College (KMC): 23RD MEETING/2019-20 dated 07/04/2020; IEC:109/2020 dated 12/02/2020; and Independent Review committee of MS Ramiah Medical College (MSRMC, Not affiliated to any of the study sites): MSRMC/EC/AP-09/06-2020). Three of our study protocols are approved as part of the single DBT-Wellcome India Alliance-funded project (Clinical Research Center for Neuromodulation in Psychiatry) and hence have the same IEC approval number.

The trial is registered in the Clinical Trial Registry of India ( CTRI/2021/05/033783) registered on 25/05/2021.

Protocol modification: Upon interim analysis or during the course of the study, any modification made to the study protocol would be reflected in the trial registries (CTRI) and would be intimated to the Ethics Committees, India Alliance CPH Committee (Funding Agency), and the journal (where the study protocol has been published).

Informed consent: Trained health professionals would obtain consent from the participants. Participants would be assessed for their capacity to consent to the study based on the capacity to consent to research studies as per the assessment using the University of California, San Diego Brief Assessment of Capacity to Consent (UBACC). Only such patients who can understand the implications of taking part in the trial would be recruited.

Family members would be involved in the consenting process, but the participants themselves would make the final decision about participating in the study. Written informed consent would be sought again before initiating the ECT.

Additional consent would be taken to use participant data for advanced analysis, future research studies, and sharing coded and de-identified data with interested national and international researchers/collaborators following the regulatory guidelines.

### Access to data

The centralized data repository would be created and maintained at NIMHANS to archive de-identified and coded data from all three institutes. The data management committee of investigators of three institutes would review data archival, management and sharing.

### Dissemination policy

A clinical trial summary report would be prepared and provided to the Institute Ethics committee and trial registries within 12 months from the completion of the study. A final (completed) trial summary report will be submitted to the funding agency for perusal. De-identified data would be shared with the research community, and public access would be granted as per the policy of DBT- Wellcome trust India Alliance and International good research practices in strict compliance with the ethics guidelines for biomedical research in India. Encrypted data storage servers would host the multi-modal clinical and neurobiological, including genetic data of all participants. The research findings would be disseminated primarily through publication in peer-reviewed journals. Prior to the publication, research data may be presented at national and international scientific fora. The study result and interpretation as a report would be shared with all major stakeholders. The team would regularly interact with all stakeholders (psychiatrists in the community, patients and caregivers, policymakers, regulatory bodies, other psychiatry institutes, and industry) to disseminate knowledge gathered from the trial.

Publications from the trial would acknowledge that the work has been carried out by all participating institutes (NIMHANS, CIP & KMC). The authorship would align with the ethical and research publication guidelines ( Resnik *et al.*, 2016). There is no intention of using professional writers in any of these works.

## Discussion

The current study would be the first to use iTBS and tDCS in a cross-over design to evaluate their comparative efficacy, complementary utility, and their mechanistic bases. Inspired by the findings of functional imaging studies, focal non-invasive brain stimulation modalities have been targeting the left TPJ for auditory hallucinations with moderate success. The aim has been to reduce significant hyperactivity in the left TPJ, the brain region consisting of Wernicke’s area and ascribed with speech generation/perception, auditory/verbal memory, multimodal perceptual integration, and self-processing ( Curcic-Blake *et al.*, 2015; Hari *et al.*, 2023; Jardri *et al.*, 2011a; Vercammen *et al.*, 2010). A recent meta-analysis comparing the efficacy of tDCS and rTMS failed to demonstrate any differential efficacy ( Guttesen *et al.*, 2021). Also, only a third of the patients to half of the patients with auditory hallucinations respond to these treatments when offered alone. The possibility of differential individual-level effectiveness cannot be ruled out. Different clinical and neurobiological factors might predict response to the iTBS and tDCS. Information on the differential mechanistic effect of these therapeutic modalities would facilitate identifying and validating such predictive factors.

Emerging evidence also suggests a higher dose, higher frequency, and longer duration of the magnetic and electrical stimulation have a better therapeutic effect. The doses/frequencies should overcome the marginal differences due to the inter-individual variations. The current protocol, thus, utilizes relatively higher cumulative doses within the well-evaluated safety standard limits.

The design of the study is also favourable to patients, where they would receive evidence-based interventions without losing precious time and other resources or additional burdens. The neuromodulatory intervention would be provided as an add-on to the ongoing pharmacotherapy and psychosocial interventions but without adding any other new intervention. This would replicate the natural clinical care settings.

We are hopeful that the result of this trial will be able to add evidence base for an informed choice of neuromodulation treatment in patients with schizophrenia with persistent auditory hallucinations.

### Declaration of interests

None of the researchers/investigators affiliated with this project or clinical research centre have any financial or non-financial (personal, academic, or political) conflict of interest to declare.

## Ethics and consent

Requisite clearances have been obtained from the ethics committees of all three institutes (i.e., NIMHANS, CIP, KMC) and from an independent ethics committee not affiliated with any of the three institutes (MS Ramaiah Medical College Ethics committee). (IEC approval numbers: Institute ethics committee of Central Institute of Psychiatry (CIP) approval: No.IEC/CIP/2020-21/337 dated 22/05/2021; Behavioural sciences division of National Institute of Mental Health and Neurosciences (NIMHANS) Institute Ethics committee approval: NIMHANS/EC (BEH.SC.DIV.) Institute ethics committee of Kasturba Medical College (KMC): 23RD MEETING/2019-20 dated 07/04/2020; IEC:109/2020 dated 12/02/2020; and Independent Review committee of MS Ramiah Medical College (MSRMC, Not affiliated to any of the study sites): MSRMC/EC/AP-09/06-2020). Three of our study protocols are approved as part of the single DBT-Wellcome India Alliance-funded project (Clinical Research Center for Neuromodulation in Psychiatry) and hence have the same IEC approval number.

Trained health professionals would obtain consent from the participants. Participants would be assessed for their capacity to consent to the study based on the capacity to consent to research studies as per the assessment using the University of California, San Diego Brief Assessment of Capacity to Consent (UBACC). Only such patients who can understand the implications of taking part in the trial would be recruited. Family members would be involved in the consenting process, but the participants themselves would make the final decision about participating in the study. Written informed consent would be sought again before initiating the ECT.

Additional consent would be taken to use participant data for advanced analysis, future research studies, and sharing coded and de-identified data with interested national and international researchers/collaborators following the regulatory guidelines.

## Data Availability

No data are associated with this article. Open Science Framework: Semi-structured proforma for “Identification of resting brain functional connectivity markers of response to continuous Theta Burst Stimulation and cathodal transcranial Direct Current Stimulation in patients with schizophrenia with persistent auditory hallucinations”. https://doi.org/10.17605/OSF.IO/HYZCT ( Sreeraj *et al.*, 2023). This project contains the following extended data: **Supplementary file 1**: 2-Consent-StudyProject-2.pdf (Informed consent form for patients) **Supplementary file 2**: Semi Structured Proforma_Study2_tDCSvsTMS in AVH.pdf Open Science Framework: SPIRIT checklist for ‘Identification of resting brain functional connectivity markers of response to continuous Theta Burst Stimulation and cathodal transcranial Direct Current Stimulation in patients with schizophrenia with persistent auditory hallucinations’. https://doi.org/10.17605/OSF.IO/HYZCT ( Sreeraj *et al.*, 2023). Data are available under the terms of the Creative Commons Zero "No rights reserved" data waiver (CC0 1.0 Public domain dedication).
