# Supplementary material for: Study protocol for identifying resting brain functional connectivity markers of response to continuous Theta Burst Stimulation and cathodal transcranial Direct Current Stimulation in patients with schizophrenia with persistent auditory hallucinations
Source: Wellcome Open Res. 2025 Feb 12;9:137. Originally published 2024 Mar 8. [Version 2] doi: 10.12688/wellcomeopenres.20956.2 (PMC12447071; doi:10.12688/wellcomeopenres.20956.2)
Supplement: Peer review history

# Reviewer report: Reviewer response for version 2

**DOI:** 10.21956/wellcomeopenres.25326.r132431

**Published:** 2025-09-18

**Authors:** Shubhmohan Singh

**Affiliations:** 1 Postgraduate Institute of Medical Education and Research, Chandigarh, India

The following points may be considered. 

 Rationale: The authors may consider justifying how the tDCS and TBS arms may differ considering the possible overlap between the mechanisms of action of both modalities.  

 Methodology: Why cross-over only the non-responders?

 What if the majority is non-responsive after both treatments?

 The authors should acknowledge that brain connectivity biomarker approach is not without limitations.

Is the study design appropriate for the research question?

Yes

Is the rationale for, and objectives of, the study clearly described?

Yes

Are sufficient details of the methods provided to allow replication by others?

Yes

Are the datasets clearly presented in a useable and accessible format?

Yes

Reviewer Expertise:

Neuromodulation, clinical adult psychiatry

I confirm that I have read this submission and believe that I have an appropriate level of expertise to confirm that it is of an acceptable scientific standard.

# Reviewer report: Reviewer response for version 2

**DOI:** 10.21956/wellcomeopenres.25326.r118578

**Published:** 2025-02-21

**Authors:** Prashanth Mayur

**Affiliations:** 1 Psychiatry, Cumberland Hospital (Ringgold ID: 60085), Westmead, New South Wales, Australia; 2 University of Sydney, Sydney, New South Wales, Australia

Thank you for the revised manuscript. I do not have any more comments to make.

Is the study design appropriate for the research question?

Yes

Is the rationale for, and objectives of, the study clearly described?

Yes

Are sufficient details of the methods provided to allow replication by others?

Partly

Are the datasets clearly presented in a useable and accessible format?

Not applicable

Reviewer Expertise:

ECT, schizophrenia, mood disorders, t DCS.

I confirm that I have read this submission and believe that I have an appropriate level of expertise to confirm that it is of an acceptable scientific standard.

# Reviewer report: Reviewer response for version 1

**DOI:** 10.21956/wellcomeopenres.23184.r81566

**Published:** 2024-05-22

**Authors:** Paul M Briley

**Affiliations:** 1 University of Nottingham, Nottingham, UK

The authors provide an RCT study protocol focused on identifying brain imaging markers of response to two alternative forms of minimally-invasive brain stimulation (neuromodulation) for persistent auditory hallucinations in people with schizophrenia. They will also collect a range of other measures that should provide a rich dataset to try to understand these difficulties that can be associated with a large amount of suffering. I have several queries, primarily around neuromodulation rationale/methodology:

 1. Note that the phrase "cathodal transcranial direct current stimulation" is not meaningful without specifying where the cathode and anode are. You plan to place the cathode over left TPJ and the anode over left DLPFC. Therefore, your direct current stimulation is "cathodal" over the left TPJ, but "anodal" over the left DLPFC. Current always flows between electrodes and both electrodes always need to be considered.

 2. Please provide details of current flow modelling to justify your targeting approaches if possible

 3. There is literature for iTBS showing that inter-run interval is very important (too short and the effects do not accumulate across runs). Is there a justification for the use of five-minute intervals between cTBS 600-pulse runs?

 4. 120% rMT is quite high intensity for cTBS. Traditional protocols use 70% or 80% rMT (or even active motor threshold). You provide justification for trying this higher intensity. However, it will certainly effect tolerability. In addition, there is likely a non-linear relationship between intensity and efficacy with TBS protocols.

 5. Just for absolute clarity, is 2 mA peak-to-peak intensity (i.e., anode at + 1 mA and cathode at - 1 mA)?

 6. You are planning to use im-TBS as a sham. Clearly, this is not sham stimulation but an active control. It is a relatively strong comparator though.

 7. Just clarify that "two such sessions will be delivered each day, spaced across at least 3 hours" applies to both of your conditions and not just the im-TMS condition

 8. Concomitant care: There may be cases where new interventions will be clinically necessary during the study. Therefore, they cannot be ruled out. Please amend this and indicate how you would manage such situations.

 9. Also, if participants have a change in antipsychotic dose of more than 25%, will they be allowed to continue with the stimulation protocol? How will you manage cases where participants are withdrawn in your data analysis? Will you perform a "worst case" analysis - there is always a risk that your approach could worsen, rather than help, symptoms, so withdrawals need to be captured.

 10. Please indicate how potential participants will be approached

 11. How will issues of capacity to consent to treatment be addressed? Will all participants have to be judged to have capacity? What if capacity is felt to be lost during the stimulation course?

 12. You indicate that TMS/t-DCS operatives will be blinded - how? (It is easy to distinguish cTBS from im-TBS from their sound)

 13. Please indicate the duration of the rsfMRI scan

 14. Will you have sufficient data to perform exploratory association analyses, and subsequent prediction analyses (should be in independent samples)?

 15. How often will the data safety monitoring board meet?

 16. Will investigators be blinded to group allocation until study completion?

 17. Safety and harm: "Very rarely, some people may feel uncomfortable". Realistically, some people find TMS (and tDCS) uncomfortable and sometimes painful. It isn't very rare. You have already considered reductions in intensity, small location adjustments could also be helpful

 18. During tDCS, will impedance monitoring be performed (high impedance is a risk factor for skin damage)?

 19. During TMS, will earplugs be worn?

 20. Were people with lived experience of schizophrenia involved in protocol development / will they be involved in trial oversight or guidance?

 21. "Written informed consent would be sought again before initiating the ECT" (is "ECT" a typo here?)

 22. Please ensure that at least patient material (e.g., Participant Information Sheet, Consent Form, videos) have been reviewed by people with lived experience of Schizophrenia and relatives. Some of the language in the PIS could be considered too technical. Please also note that there is a risk that you could make their experiences worse, this is always a possibility.

 23. The PIS (near the start) also refers to stimulation lasting three minutes - indicate the true length of the protocol.

 24. Please indicate, in the PIS, how withdrawals (by the experimenters due to new medications etc.) from the study will be managed

 25. To what extent will there be sharing of the rich dataset generated by this study, to support analyses by the wider research community?

Is the study design appropriate for the research question?

Partly

Is the rationale for, and objectives of, the study clearly described?

Yes

Are sufficient details of the methods provided to allow replication by others?

Partly

Are the datasets clearly presented in a useable and accessible format?

Not applicable

Reviewer Expertise:

Neuromodulation techniques - TMS and tES; Psychiatry

I confirm that I have read this submission and believe that I have an appropriate level of expertise to confirm that it is of an acceptable scientific standard, however I have significant reservations, as outlined above.

# Reviewer report: Reviewer response for version 1

**DOI:** 10.21956/wellcomeopenres.23184.r79692

**Published:** 2024-05-16

**Authors:** Prashanth Mayur

**Affiliations:** 1 Psychiatry, Cumberland Hospital (Ringgold ID: 60085), Westmead, New South Wales, Australia; 2 University of Sydney, Sydney, New South Wales, Australia

**Summary**

 It is a well written manuscript, methodologically rigorous and ambitious in scope. The authors may be attempting a paradigm shift in clinical practice by treating persistent auditory hallucinations in schizophrenia, inspite of *one *antipsychotic treatment exposure, with newer brain stimulation approaches (c t DCS or Theta Burst TMS). This approach is clinically appropriate and is to be commended in view of the relative non-invasive nature of these interventions vis a vis ECT. The large sample size and the rigorous methodology may allow the outcomes to have translational value in clinical psychiatry. In addition, the study may bring out new findings, of the efficacy of the interventions being related to differential states of resting state functional connectivity while producing similar/equivalent clinical response of ameliorating auditory verbal hallucinations, thus adding value to basic science research. 

 There are some minor points of revision that may tighten up the methods.

 Introduction

 Introduction is generally well written.

 - In paragraph 2, a brief introductory line on r TMS similar to the introduction for t DCS is useful for lay readers and general psychiatrists.

 -A brief write-up is useful about the rationale for choosing the timing of neurostimulatory interventions after a minimum cut off of one antipsychotic treatment failure. 

 Aims and Hypothesis

 - In the primary hypothesis, "to identify resting brain functional connectivity markers....."

 It is not clear to me if the broad approach is a seed based approach or a data driven approach. If it is a seed based approach, please clarify putative brain regions. Either way, it is recommended that there is some clarity on this matter in the preliminary paper, although I am aware that the authors are planning to write a separate paper on this matter. 

 Selection of patients

 -Are you excluding patients who have received ECT in the recent past. It will be useful to make this clear apart from excluding patients who may require ECT due to a clinical emergency.

 - From a purist perspective, you have kept a traditional diagnostic framework of schizophrenia and are exploring the impact of interventions on auditory verbal hallucinations in schizophrenia. However, from a perspective of a continuum of psychosis, it is disappointing that a large number of patients with auditory hallucinations related to other non affective psychosis or multi-episodic methamphetamine psychosis will be excluded. These are 'orphan categories' with hardly any research and a large illness burden. With the large number of patients potentially recruited in this study, I would have preferred a broader approach to examine auditory verbal hallucinations extending up to non affective psychosis and including substance induced persistent auditory hallucinations.

 - It will be useful to be precise about post-partum state.

 Interventions. Interventions and the cross-over design are clearly spelt out. The ensuring of an 'active treatment arm irrespective of the sham treatment' is ethically sound. A lot of effort has gone into ensuring appropriate blinding. Plan for data management and analysis  are thorough and sophisticated. Sample size determination is thorough and conservative.

 A few minor revisions may be in order.

 - Please expand AC-PC plane.

 - within participant timeline, the sentence- "Participants with <30% improvement.... 2-4 weeks.....". Please clarify when exactly AHRS will be conducted after step 1 to determine non-response. 

 -If there is non-response, the washout period of 2-4 weeks is inconsistent and rather lengthy. Does this mean that some may have a washout for 2 weeks and others for 3 or 4 weeks?. Please justify this washout period. 

 -For emergency unblinding, please clarify *who* will report the SAE to the trial management group and the data safety board (eg- principle investigator and or co-investigators).

 Discussion

 A comment

 - The intent of the cross over design to compare c TDCS and theta burst TMS is laudable and a novelty. It appears that while efforts have been made to optimise TMS at 120% RMT with 1800 pulses, c TDCS is still traditional (2mA for 20 minutes). This is only an observation and may not influence clinical outcomes. An analogy may be apt: while apples may be compared with oranges, both may be equally capable of satisfying hunger.

Is the study design appropriate for the research question?

Yes

Is the rationale for, and objectives of, the study clearly described?

Yes

Are sufficient details of the methods provided to allow replication by others?

Partly

Are the datasets clearly presented in a useable and accessible format?

Not applicable

Reviewer Expertise:

ECT, schizophrenia, mood disorders, t DCS.

I confirm that I have read this submission and believe that I have an appropriate level of expertise to confirm that it is of an acceptable scientific standard, however I have significant reservations, as outlined above.
